# Associations Between Vitamin D Levels and Depressive Symptoms in Later Life: Evidence From the English Longitudinal Study of Ageing (ELSA)

**DOI:** 10.1093/gerona/glx130

**Published:** 2017-06-22

**Authors:** Cesar de Oliveira, Vasant Hirani, Jane P Biddulph

**Affiliations:** 1 Department of Epidemiology & Public Health, English Longitudinal Study of Ageing (ELSA), University College London (UCL), UK; 2 Nutrition and Dietetics Group, School of Life and Environmental Sciences Charles Perkins Centre, University of Sydney, New South Wales, Australia; 3 ARC Centre of Excellence in Population Ageing Research, University of Sydney, New South Wales, Australia

**Keywords:** 25-hydroxyvitamin D, Depression, Older adults, Ageing

## Abstract

**Background:**

A possible role of vitamin D in depression has received considerable attention, especially given the significant disability, mortality, and healthcare costs associated to depression and the high prevalence of vitamin D deficiency.

**Methods:**

We investigated the cross-sectional associations between serum 25-hydroxyvitamin D (25OHD) levels and depressive symptoms (CES-D) in 5,607 older adults from the English Longitudinal Study of Ageing (ELSA).

**Results:**

Overall, there was a significant association between low 25OHD levels and elevated depressive symptoms (odds ratio [OR] = 1.58, 95% confidence interval [CI] = 1.20–2.07 for the lowest quartile; OR = 1.45, 95% CI = 1.15–1.83 for <30 nmol/L cut-off and OR = 1.34, 95% CI = 1.10–1.62 for the ≤50 nmol/L cut-off) after adjustment for a wide range of covariates of clinical significance. Fully adjusted models showed that women in the lowest (OR = 1.67, 95% CI = 1.20–2.34) and second lowest (OR = 1.68, 95% CI = 1.20–2.35) quartiles of 25OHD as well as those with 25OHD levels <30 nmol/L (OR = 1.40, 95% CI = 1.06–1.86) and ≤50 nmol/L (OR = 1.35, 95% CI = 1.07–1.72) were more likely to report elevated depressive symptoms. For men, however, this association only remained significant for those with 25OHD levels of <30 nmol/L (OR = 1.60, 95% CI = 1.06–2.42) in the fully adjusted models.

**Conclusions:**

The independent and inverse association found between low 25OHD levels and elevated depressive symptoms suggests that vitamin D deficiency may be a risk factor for late-life depression, particularly among women. Whether our findings have any clinical meaning or not, additional data are needed from well-designed randomized controlled trials of vitamin D for the prevention and treatment of late-life depression.

Depression is associated with significant disability, mortality, and high healthcare costs (1). It is responsible for between 5% and 8% of the total disability-adjusted life years (DALYs) in middle and high-income countries (2). Despite its high prevalence, however, depression among older adults tends to be under-recognized and under-treated (3). Although the underlying pathophysiology of depression remains unknown, recent advances in basic and clinical research highlighted the potential role of new biological factors that may affect mood in combination with the more traditional neurochemical and neuroendocrine mechanisms (4).

Low vitamin D status is a major public health problem worldwide, particularly in older people (5). It is projected that about 1 billion people globally have vitamin D deficiency or insufficiency (6). The serum concentrations of 25-hydroxyvitamin D (25OHD), the major storage and circulating form of vitamin D rises and falls with the supply of vitamin D3 (cholecalciferol) and vitamin D2 (ergocalciferol). Older adults are at increased risk of poor vitamin D status due to the lack of sun exposure and to an age-related decline in the efficiency of vitamin D synthesis and metabolism (7). The importance of vitamin D in the absorption and metabolism of calcium for bone health is well established (8). Besides its effect on calcium metabolism and bone health, vitamin D deficiency has been linked to various diseases and proposed to be a universal risk factor for multiple multifactorial diseases. For example, studies demonstrated that low 25OHD concentrations may promote the pathogenesis of type 1 diabetes (9), rheumatoid arthritis (10), multiple sclerosis (11), sarcopenia (12), cancer (13), and other diseases. Other actions of vitamin D include its impact on the innate and adaptive immunity (14).

A possible role of vitamin D in depression has received considerable attention. To date epidemiological evidence from several cross-sectional studies have shown an association of vitamin D deficiency with an increase in depression (15). Very few longitudinal studies, with conflicting findings, have prospectively explored the relationship between vitamin D deficiency and late-life depression (16,17). Evidence from recently published systematic reviews and meta-analyses (15,18–20) identified an overall significant association between depression and vitamin D deficiency. However, nonsignificant findings from another meta-analysis have been reported (21). Furthermore, causality and efficacy of supplementation remain controversial.

Given the high prevalence of both vitamin D deficiency and depression, an association between these two conditions would have significant public health and clinical implications. The evidence for older adults from the general population in England is scarce and outdated. Therefore, the primary objective of this cross-sectional analysis was to investigate the association between the 25OHD concentrations and depressive symptoms, using data from the English Longitudinal Study of Ageing (ELSA). ELSA was designed to be nationally representative of community dwelling adults aged 50 years and older from the general population of England, United Kingdom.

## Methods

### Study Population

ELSA is an ongoing prospective observational study of community-dwelling people aged 50 years and over in England that commenced in 2002. The ELSA sample was drawn from participants that had previously participated in the Health Survey for England (HSE); an annual health examination survey, which each year recruits a different nationally representative sample using a multi-staged stratified random probability design (22). After baseline, follow-up interviews within ELSA occur every 2 years and health examinations, that is, a nurse visit, every 4 years. The first health examination was in 2004–2005. A detailed description of the study can be found elsewhere (23). Analyses for this study used cross-sectional data from wave 6 (2012–2013) as this was the first time that 25OHD concentrations were ascertained in ELSA.

### Assessment of 25OHD

Wave 6 had 10,601 respondents that also included noncore members, such as, partners. Only the 9,169 core sample members eligible for a nurse visit at which blood samples could be taken were included. Of those, 7,730 had a nurse visit. Blood samples were obtained from 6,206 participants, and 25OHD concentrations were ascertained in 5,870. Blood samples were not taken from those who had a clotting or bleeding disorder (eg, haemophilia or low platelets), had ever had a fit, were currently on anticoagulant drugs (eg, warfarin therapy) or were did not give their consent in writing. The analyses of blood samples were carried out at the Royal Victoria Infirmary (Newcastle-upon-Tyne, United Kingdom). Serum 25OHD levels were measured by the Diasorin Liaison immunoassay that detects both 25OHD2 and 25OHD3 and therefore, provides the total circulating 25OHD level, as previously described (21). The assay for 25OHD has an analytical sensitivity (lower detection limit) of 7.5 nmol/L. The detection limit represents the lowest measurable analyte level that can be distinguished from zero. All assays were performed in duplicate. The coefficient of variation ranged from 8.7% to 9.4%. The laboratory performing the 25OHD analyses took part in the Internal and the Vitamin D External Quality Assessment Schemes (DEQAS).

### Assessment of Depressive Symptoms

Depressive symptomatology was measured using the eight-item Center for Epidemiological Studies-Depression (CES-D) scale, a widely used self-report measure of depressive symptoms. CES-D is not a diagnostic instrument for clinical depression but can be used to identify people “at risk” of depression in population-based studies (24). This short version had good internal consistency at each wave (Cronbach’s α > .95) and comparable psychometric properties to the full 20-item CES-D (25,26). Five of the eight CES-D items (ie, felt depressed, was happy, felt lonely, enjoyed life, felt sad) were depressed mood items, while the remaining three (ie, everything was an effort, restless sleep, and could not get going) were somatic complaints items (25). We derived a summary CES-D score by adding responses to all eight dichotomous questions (possible range: 0–8). To identify cases of elevated depressive symptoms that are possible cases of clinical depression we dichotomized the summary score around the cut point of four or greater (25,26). This definition has been validated against standardized psychiatric interviews in older populations (26).

### Covariates

Total non-pension household wealth included financial wealth (savings and investments), the value of any home and other property (less mortgage), the value of any business assets and physical wealth such as artwork and jewellery, net of debt. Wealth is the most robust indicator of socioeconomic circumstances in ELSA, and has been found to be more strongly associated with the risk of death than any other socioeconomic position indicator at older ages (27). The number of comorbidities, that is, cardiovascular disease and other chronic conditions was assessed by self-reported doctor diagnosed chronic diseases that included diabetes, cancer, stroke, arthritis, lung disease, and Parkinson’s and for cardiovascular diseases included high blood pressure, angina, heart attack, heart failure, heart murmur or heart rhythm. Smoking status was classified into nonsmokers, former smokers, or current smokers. Self-reported physical activity included questions about the frequency of participation in vigorous, moderate, and mild physical activities; more than once per week, once per week, one to three times per month, hardly ever (sedentary). Physical activity was further categorized into four groups: sedentary; low; moderate and vigorous. Physical functioning was measured using self-reported limitations in the following basic Activities of Daily Living (ADL): dressing, walking across a room, bathing or showering, eating, getting in or out of bed, using the toilet. Self-reported limitations in the following Instrumental Activities of Daily Living (IADL): making telephone calls, shopping for groceries, preparing hot meal, doing work around the house or garden, taking medications, and managing money, such as paying bills and keeping track of expenses were also measured. A physical functioning limitation was defined as having a limitation in one or more ADL or IADL activities. A total memory score was measured using a word-list learning test, in which 10 words were presented orally to study participants who were then asked to recall as many as possible immediately after reading them and then again after a 5-minute delay during which they completed other survey questions. We computed an overall memory score (range, 0–20) using both the immediate and delayed recall results (between-test correlation coefficient = .70). Waist circumference was categorized into three main groups using sex-specific cut-offs: low (<94 cm for men and <80 cm for women), medium (≥94 cm and <102 cm for men; ≥80 cm and <88 cm for women) and high (≥102 cm for men and ≥88 cm for women).

### Statistical Analyses

A complete case analysis with a final analytical sample of 5,607 participants was performed. For stratified analyses by sex, there was a final analytical sample of 2,525 males, and 3,082 females. Whilst there was an absence of evidence of age or sex interactions with 25OHD quartiles, sex interactions were both apparent with other covariates used in the model and with 25OHD cut-offs. Logistic regression was used to investigate the unadjusted and adjusted association between 25OHD levels with depressive symptoms. Due to the lack of international consensus (28), 25OHD levels, the independent variable, was categorized into quartiles: the lowest quartile (≤30 nmol/L), second quartile (30.01 until 46.00 nmol/L), third quartile (46.01 until 64.00 nmol/L) and the highest quartile (>64.01 nmol/L; reference category). We have also categorized 25 OHD levels using the cut-off points recommended by the US Institute of Medicine (IOM) (29): <30 nmol/L; 30–50 nmol/L; >50 nmol/L. The UK National Osteoporosis Society (NOS 2013) recently proposed that IOM vitamin thresholds should be adopted by UK practitioners. A truncated categorization of ≤50 nmol/L and >50 nmol/L was also used (6). Following the unadjusted model (model 1), a further five models (models 2 to 6) were derived to investigate the association between 25OHD with depression, and adjusted for potential confounders. Each subsequent model included the variables that were included in the previous model. Model 2, adjusted for age, sex, and the season of the blood sampling. Model 3, further adjusted for wealth. Model 4 further adjusted for health behaviors; smoking and physical activity. Model 5 further adjusted for health; number of cardiovascular conditions, number of non-cardiovascular chronic conditions, difficulties in ADL, difficulties in IADL and total memory score. Finally, model 6 further adjusted for waist circumference. The analyses were performed using STATA 13.0 (Stata Corp LLP, College Station, TX).

### Ethics Approval and Informed Consent

All participants gave written informed consent. The National Research Ethics Service (London Multicentre Research Ethics Committee [MREC/01/2/91]) has approved the ELSA.

## Results

Baseline characteristics of the sample for which 25OHD levels were ascertained by depression status and by gender can be found in the Supplementary Tables 1 and 2, respectively. Forty-two percent of the blood samples were taken in Autumn. Twelve percent reported depressive symptoms, and 61% and 56% reported cardiovascular and non-cardiovascular chronic conditions, respectively. The majority did not report difficulties in performing either basic (85%) or instrumental (83%) ADL. 25OHD levels ranged between 9 and 239 nmol/L and the mean was 48.70 nmol/L (*SD* = 23.57 nmol/L). The mean total memory score was 11 (*SD* = 3.5) and half of the participants had a high waist circumference. Those for whom 25OHD data were not available compared with those for whom data were available were older and reported more ADL and IADL disability, more cardiovascular disease, depressive symptoms, sedentary lifestyle, and lower level of wealth (*p* < .05 data not shown).

In the unadjusted analyses, low serum levels of 25OHD were associated with elevated depressive symptoms (Tables 1 and 2). The lowest and the second-lowest quartiles of 25OHD (odds ratio [OR] = 2.40, 95% confidence interval [CI] = 1.89–3.04; OR = 1.58, 95% CI = 1.23–2.03; Table 1, model 1) as well as 25OHD levels <30 nmol/L, 30–50 nmol/L and ≤50 nmol/L (OR = 2.26, 95% CI = 1.85–2.76; OR=1.35, 95% CI = 1.10–1.64; OR=1.71, 95% CI = 1.44–2.03; Table 2, model 1) were significantly associated with higher levels of depressive symptoms in the unadjusted analysis. This association remained statistically significant even after the adjustment for all covariates included in the models but with attenuation in the OR from model 2 to model 6 (Tables 1 and 2). For women, those in the lowest (OR = 2.17, 95% CI = 1.60–2.93) and second lowest (OR = 1.69, 95% CI = 1.23–2.32) quartiles of 25OHD as well as those with 25OHD levels <30 nmol/L (OR = 1.92, 95% CI = 1.50–2.47), 30–50 nmol/L (OR = 1.38, 95% CI = 1.07–1.77) and ≤50 nmol/L (OR = 1.61, 95% CI = 1.30–2.00) were more likely to report elevated depressive symptoms. This association remained statistically significant even after adjustment for all covariates included in the models but with less marked decline in OR from model 2 to model 6 (Table 1 and 2). For men, however, the only association that remained significant in the fully adjusted model was for those with 25OHD levels <30 nmol/L (OR = 1.60, 95% CI = 1.06–2.42; Table 2, model 6).

**Table 1.** Multivariable Logistic Regression Analyses Investigating the Association Between Depression and 25-Hydroxy Vitamin D (25OHD) Concentrations

	Model 1 (unadjusted)	Model 2	Model 3	Model 4	Model 5	Model 6
	OR (95% CI)	OR (95% CI)	OR (95% CI)	OR (95% CI)	OR (95% CI)	OR (95% CI)
All participants^a^						
25OHD						
Highest quartile	1	1	1	1	1	1
3rd quartile	1.16 (0.89–1.52)	1.18 (0.90–1.53)	1.12 (0.86–1.47)	1.15 (0.88–1.51)	1.19 (0.90–1.57)	1.20 (0.91–1.59)
2nd quartile	1.58 (1.23–2.03)	1.60 (1.24–2.06)	1.41 (1.09–1.83)	1.44 (1.11–1.87)	1.46 (1.12–1.91)	1.49 (1.13–1.95)
Lowest quartile	2.40 (1.89–3.04)	2.39 (1.87–3.07)	1.80 (1.39–2.32)	1.65 (1.27–2.14)	1.55 (1.18–2.02)	1.58 (1.20–2.07)
Males						
25OHD						
Highest quartile	1	1	1	1	1	1
3rd quartile	1.03 (0.66–1.61)	1.01 (0.64–1.59)	0.93 (0.59–1.47)	0.92 (0.58–1.46)	0.95 (0.59–1.55)	0.94 (0.58–1.53)
2nd quartile	1.42 (0.93–2.18)	1.46 (0.95–2.25)	1.24 (0.80–1.92)	1.16 (0.74–1.81)	1.24 (0.77–1.98)	1.21 (0.75–1.94)
Lowest quartile	2.75 (1.85–4.08)	2.91 (1.90–4.44)	1.92 (1.24–2.98)	1.61 (1.03–2.51)	1.47 (0.92–2.36)	1.44 (0.90–2.31)
Females						
25OHD						
Highest quartile	1	1	1	1	1	1
3rd quartile	1.26 (0.91–1.75)	1.27 (0.92–1.77)	1.22 (0.88–1.71)	1.29 (0.92–1.81)	1.32 (0.94–1.86)	1.38 (0.98–1.95)
2nd quartile	1.69 (1.23–2.32)	1.69 (1.23–2.32)	1.52 (1.10–2.09)	1.60 (1.15–2.12)	1.58 (1.13–2.19)	1.68 (1.20–2.35)
Lowest quartile	2.17 (1.60–2.93)	2.16 (1.58–2.94)	1.72 (1.25–2.36)	1.66 (1.20–2.28)	1.57 (1.13–2.18)	1.67 (1.20–2.34)

*Notes*: CI = confidence interval; OR = odds ratio. Model 1: 25OHD (unadjusted); Model 2: Model 1 + adjusted for sex^a^, age group, and season; Model 3: Model 2 + adjusted for wealth; Model 4: Model 3 + adjusted for smoking, and physical exercise; Model 5: Model 4 + adjusted for number of cardiovascular conditions, number of non-cardiovascular conditions, difficulties in activities of daily living, difficulties in instrumental activities of daily living, and total memory score; Model 6: Model 5 + adjusted for waist circumference.

^a^Models 2 to 6 also adjusted for sex.

**Table 2.** Multivariable Logistic Regression Analyses Investigating the Association Between Depression and 25-Hydroxy Vitamin D (25OHD) by Classification System

	Model 1 (unadjusted)	Model 2	Model 3	Model 4	Model 5	Model 6
25OHD	OR (95% CI)	OR (95% CI)	OR (95% CI)	OR (95% CI)	OR (95% CI)	OR (95% CI)
IOM cut points (29)						
All participants^a^						
>50 nmol/L	1	1	1	1	1	1
30–50 nmol/L	1.35 (1.10–1.64)	1.36 (1.11–1.67)	1.24 (1.01–1.53)	1.23 (1.00–1.52)	1.24 (1.00–1.54)	1.26 (1.01–1.56)
<30 nmol/L	2.26 (1.85–2.76)	2.25 (1.82–2.77)	1.72 (1.39–2.14)	1.55 (1.24–1.93)	1.43 (1.14–1.80)	1.45 (1.15–1.83)
Males						
>50 nmol/L	1	1	1	1	1	1
30–50 nmol/L	1.31 (0.93–1.85)	1.37 (0.96–1.94)	1.22 (0.85–1.74)	1.11 (0.78–1.60)	1.20 (0.82–1.76)	1.18 (0.80–1.73)
<30 nmol/L	2.87 (2.06–4.00)	3.09 (2.15–4.45)	2.14 (1.47–3.11)	1.76 (1.19–2.59)	1.63 (1.08–2.46)	1.60 (1.06–2.42)
Females						
>50 nmol/L	1	1	1	1	1	1
30–50nmol/L	1.38 (1.07–1.77)	1.37 (1.06–1.76)	1.25 (0.97–1.62)	1.29 (1.00–1.67)	1.27 (0.97–1.65)	1.32 (1.01–1.72)
<30 nmol/L	1.92 (1.50–2.47)	1.90 (1.47–2.47)	1.53 (1.17–2.00)	1.44 (1.10–1.89)	1.34 (1.01–1.77)	1.40 (1.06–1.86)
Truncated cut points						
All participants^a^						
>50 nmol/L	1	1	1	1	1	1
≤50 nmol/L	1.71 (1.44–2.03)	1.69 (1.41–2.02)	1.43 (1.19–1.71)	1.36 (1.13–1.64)	1.32 (1.09–1.60)	1.34 (1.10–1.62)
Males						
>50 nmol/L	1	1	1	1	1	1
≤50 nmol/L	1.88 (1.40–2.52)	1.89 (1.39–2.57)	1.53 (1.12–2.10)	1.34 (0.97–1.85)	1.36 (0.97–1.92)	1.34 (0.95–1.88)
Females						
>50 nmol/L	1	1	1	1	1	1
≤50 nmol/L	1.61 (1.30–2.00)	1.58 (1.27–1.97)	1.37 (1.09–1.71)	1.35 (1.08–1.70)	1.30 (1.03–1.64)	1.35 (1.07–1.72)

*Notes*: CI = confidence interval; IOM = Institute of Medicine; OR = odds ratio. Model 1: 25OHD (unadjusted); Model 2: Model 1 + adjusted for sex^a^, age group, and season; Model 3: Model 2 + adjusted for wealth; Model 4: Model 3 + adjusted for smoking, and physical exercise; Model 5: Model 4 + adjusted for number of cardiovascular conditions, number of non-cardiovascular conditions, difficulties in activities of daily living, difficulties in instrumental activities of daily living, and total memory score; Model 6: Model 5 + adjusted for waist circumference.

^a^Models 2 to 6 also adjusted for sex.

## Discussion

In this large population-based cohort of older adults aged 50 years and older, we demonstrated that older English adults from the general population who have low 25OHD serum levels are more likely to report elevated depressive symptoms. This study observed significant negative associations between 25OHD levels and elevated depressive symptoms especially among women. In our analyses, the association observed between low 25OHD levels and elevated depressive symptoms was independent of confounders: age, economic circumstances and a wide range of health conditions, health behaviors, physical function, and cognitive function.

Observational studies provide some evidence for a relationship between vitamin D deficiency and depression (20,30–35). Findings from the present study are consistent with other studies (20) in which increased elevated depressive symptoms were seen in individuals with low vitamin D status. Various reviews suggested an association between vitamin D and depression (30–35), and recent systematic review and meta-analysis of observational studies and randomized controlled trials concluded that low vitamin D concentration is associated with depression in adults (15,18–20).

Low vitamin D levels have been observationally associated with depression and depressive symptoms and, therefore, an antidepressant effect of vitamin D supplementation could be expected. The potential role for vitamin D in late-life depression prevention and treatment remains uncertain. A systematic review aimed to summarize the evidence of randomized clinical trials to assess the efficacy of oral vitamin D supplementation in depression compared with placebo (21) concluded that there was insufficient evidence to support the efficacy of vitamin D supplementation in depression symptoms, and more randomized clinical trials using depressed patients are warranted. In contrast, Shaffer et al found that vitamin D supplementation may be effective for reducing depressive symptoms in patients with clinically significant depression; however, further high quality research is needed (19). Similarly, a systematic review and meta-analysis comparing studies with and without biological flaws concluded that vitamin D supplementation was somewhat favorable in the management of depression in studies that demonstrate a change in vitamin levels, and the effect size was comparable to that of antidepressant medication (15,18).

It is difficult to identify the causal relation given the observational design of most studies investigating the link between vitamin D and depression and the numerous potential confounders, especially when there is reverse causality between serum vitamin level and depression. For example, depressed individuals tend to go outside less frequently and thus are typically less exposed to sunlight. In addition, their associated reduced appetite may also lead to dietary changes that then reduce their vitamin D levels.

Although the association between vitamin D and depression has not yet been confirmed, there are indications that a low vitamin D concentration may predispose to depression through several biological mechanisms. Current hypotheses about the pathophysiological mechanisms in the association between vitamin D and depression include a role for vitamin D in the regulation of the neurotransmitters dopamine, noradrenaline and acetylcholine, as well as an effect on neurotrophic factors (36). Moreover, vitamin D receptors are found in the prefrontal cortex and parts of the limbic system. These brain areas have been implicated in the pathophysiology of depression (36). Vitamin D may also indirectly fight depressive symptoms via its proposed anti-inflammatory effect (37). Another important function of vitamin D is to control the formation of serotonin and this is another feature of the link between vitamin D deficiency and depression (38). It has been shown that one of the actions of vitamin D is to induce the expression of the serotonin-synthesizing gene tryptophan hydroxylase 2 while repressing the expression of tryptophan hydroxylase 1. Both tryptophan hydroxylase 1 and tryptophan hydroxylase 2 play a role in serotonin synthesis. Vitamin D may thus prevent depression by maintaining normal serotonin levels.

Our findings showed that amongst women there was an elevated risk for depression over a broader range of vitamin D deficiency of less or equal to 50 nmol/L and in the lowest two quartiles of 25OHD in women. For men, however, an elevated risk for depression was true only for a deficiency of lower than 30 nmol/L. A higher prevalence of depression in women compared with men is consistent across nations, cultures, and population groups, in studies using different methods and measurement instruments, and for a diversity of incidence and prevalence indicators (39). Cross-national comparative research and meta-analyses usually put the gender ratio in the prevalence of depression in Western countries at approximately 2:1 (40). In addition, a higher prevalence of vitamin D deficiency in women has also been reported (16).

Given the high prevalence of both vitamin D deficiency and depression, an association between these two conditions would have significant public health implications, particularly as supplementation with vitamin D is cost-effective and without significant adverse effects. The increase of the natural production of serotonin by vitamin D could potentially enhance serotonin that is taken orally as an antidepressant. The clinical implications of our findings are that health professionals should be alerted to complaints presented to them by older people, so that appropriate interventions can be provided, as vitamin D deficiency and late-life depression are common in older people and both have adverse health consequences.

Our study has several strengths and potential limitations that need to be considered. As one of the largest samples with vitamin D levels in the United Kingdom, a major strength is the large and representative sample of community-dwelling English men and women aged 50 years and older using a wide range of covariates. In addition, certified examiners following standardized protocols, assuring excellent quality of data, performed all examinations and laboratory measurements. We have also used an internationally validated questionnaire specific for depressive symptoms. Limitations arise from the cross-sectional study design, which investigates associations but cannot provide evidence of causality. Data on repeated measures for vitamin D were not available, and therefore we could not investigate the directionality of the association, and whether depressive symptoms occurred after vitamin D deficiency. However, ELSA is planning to measure levels of vitamin D again in its eighth wave (2016–2017), which will enable longitudinal analyses utilizing these repeated measures. We were unable to assess differences between vitamin D2 and vitamin D3 as the respective data were not available in ELSA. Another potential limitation could be attributed to selection bias, in that out of the 9,169 participants eligible for a nurse visit at which blood samples could be taken, 25OHD concentrations were ascertained in 5,870. However, it is also possible that our effect estimates may be conservative since people who gave blood were more likely to be healthier than those who did not. Lack of knowledge of current antidepressant use could create a bias, although this seems unlikely to have had a substantial effect since only 3% of our population reported having used antidepressants in the last 2 years. Our findings were obtained in community-dwelling subjects mainly aged below 80 years, with low disability and might not be representative of frailer, or older subjects, and nursing home residents. Finally, reverse causality, in which depression leads to decreased sun exposure and poorer dietary intake, thereby causing vitamin D deficiency, cannot be ruled out in cross-sectional studies.

In summary, our study suggests a potential role of 25OHD in depression, especially among women. Our findings, therefore, contribute significantly to the body of evidence supporting a role for vitamin D in depression. Whether our findings have any clinical meaning or not, additional data from well-designed randomized controlled trials of vitamin D for the prevention and treatment of depression in late-life are needed. The development of strategies to prevent late-life depression is necessary to reduce its impact on disease burden and associated costs.

## Supplementary Material

Supplementary data is available at *The Journals of Gerontology, Series A: Biological Sciences and Medical Sciences* online.

## Funding

The English Longitudinal Study of Ageing is supported by the National Institute on Aging (NIA/NIH) USA (grant number 5 R01 AG017644-16) and a consortium of the United Kingdom government departments coordinated by the Economic and Social Research Council (ESRC). These funding bodies had no role in the study design; in the collection, analysis, and interpretation of data; in the writing of the report; and in the decision to submit the article for publication.

## Conflict of Interest

None reported.

## Supplementary Material

Supplementary_Tables
