# Supplementary material for: Associations Between Vitamin D Levels and Depressive Symptoms in Later Life: Evidence From the English Longitudinal Study of Ageing (ELSA)
Source: J Gerontol A Biol Sci Med Sci. 2017 Jun 22;73(10):1377–82. doi: 10.1093/gerona/glx130 (PMC6132123; doi:10.1093/gerona/glx130)
Supplement: Supplementary_Tables [file glx130_suppl_supplementary_tables.docx]

Supplementary Table 1. Characteristics in the analytical sample of 5,607 participants aged 50 years and older by depression status from the English Longitudinal Study of Ageing (2012-2013)

|  | All participants  n (%) | ‘Depression’  CES-D score 4 or more  n (%) | ‘No depression’  CES-D score <4  n (%) |
| --- | --- | --- | --- |
| **Depression*** |  |  |  |
| Yes | 657 (11.7) | 657 (11.7) | - |
| No | 4,950 (88.3) | - | 4,950 (88.3) |
| **Vitamin D quartile** |  |  |  |
| Lowest | 1,406 (25.1) | 244 (37.1) | 1,255 (25.4) |
| Second lowest | 1,400 (25.0) | 170 (25.9) | 1,303 (26.3) |
| Second highest | 1,436 (25.6) | 133 (20.2) | 1,230 (24.9) |
| Highest | 1,365 (24.3) | 110 (16.7) | 1,162 (23.5) |
| **IOM^ꝉ^ vitamin D (25OHD) cut points** |  |  |  |
| >50nmol/L | 2,435 (43.4) | 212 (32.3) | 2,223 (44.9) |
| 30-50nmol/L | 1,847 (32.9) | 210 (32.0) | 1,637 (33.1) |
| <30nmol/L | 1,325 (23.6) | 235 (35.8) | 1,090 (22.0) |
| **Truncated vitamin D (25OHD) cut points** |  |  |  |
| >50nmol/L | 2,435 (43.4) | 212 (32.3) | 2,223 (44.9) |
| ≤50nmol/L | 3,172 (56.6) | 445 (67.7) | 2,727 (55.1) |
| **Sex** |  |  |  |
| Male | 2,525 (45.0) | 229 (34.9) | 2,296 (46.4) |
| Female | 3,082 (55.0) | 428 (65.1) | 2,654 (53.6) |
| **Age group** |  |  |  |
| 50-59 years | 1,302 (23.2) | 202 (30.8) | 1,100 (22.2) |
| 60-69 years | 2,283 (40.7) | 223 (33.9) | 2,060 (41.6) |
| 70-79 years | 1,491 (26.6) | 157 (23.9) | 1,334 (27.0) |
| 80+ years | 531 (9.5) | 75 (11.4) | 456 (9.2) |
| **Season of blood collection** |  |  |  |
| Winter | 1,482 (26.4) | 180 (27.4) | 1,302 (26.3) |
| Spring | 416 (7.4) | 52 (7.9) | 364 (7.4) |
| Summer | 1,324 (23.6) | 157 (23.9) | 1,167 (23.6) |
| Autumn | 2,385 (42.5) | 268 (40.8) | 2,117 (42.8) |
| **Wealth quintile** |  |  |  |
| Lowest quintile | 821 (14.6) | 207 (31.5) | 614 (12.4) |
| 2^nd^ quintile | 1,041 (18.6) | 156 (23.7) | 885 (17.9) |
| 3^rd^ quintile | 1,200 (21.4) | 128 (19.5) | 1,072 (21.7) |
| 4^th^ quintile | 1,252 (22.3) | 89 (13.6) | 1,163 (23.5) |
| Highest quintile | 1,293 (23.1) | 77 (11.7) | 1,216 (24.6) |
| **Smoking status** |  |  |  |
| Non-smoker | 2,156 (38.5) | 203 (30.9) | 1,953 (39.5) |
| Former smoker | 2,822 (50.3) | 298 (45.4) | 2,524 (51.0) |
| Current smoker | 629 (11.2) | 156 (23.7) | 473 (9.6) |
| **Physical activity** |  |  |  |
| Sedentary | 226 (4.0) | 72 (11.0) | 154 (3.1) |
| Low | 812 (14.5) | 186 (28.3) | 626 (12.7) |
| Moderate | 2,729 (48.7) | 289 (44.0) | 2,440 (49.3) |
| High | 1,840 (32.8) | 110 (16.7) | 1,730 (35.0) |
| **Number of cardiovascular conditions** |  |  |  |
| None | 2,166 (38.6) | 199 (30.3) | 1,967 (39.7) |
| 1 | 1,695 (30.2) | 190 (28.9) | 1,505 (30.4) |
| 2+ | 1,746 (31.1) | 268 (40.8) | 1,478 (29.9) |
| **Number of non-cardiovascular chronic conditions** |  |  |  |
| None | 2,459 (43.9) | 161 (24.5) | 2,298 (46.4) |
| 1 | 2,094 (37.4) | 241 (36.7) | 1,853 (37.4) |
| 2+ | 1,054 (18.8) | 255 (38.8) | 799 (16.4) |
| **Difficulties in activities of daily living (ADL)** |  |  |  |
| None | 4,779 (85.2) | 421 (64.1) | 4,358 (88.0) |
| 1+ | 828 (14.8) | 236 (35.9) | 592 (12.0) |
| **Difficulties in instrumental activities of daily living (IADL)** |  |  |  |
| None | 4,666 (83.2) | 386 (58.8) | 4,280 (86.5) |
| 1+ | 941 (16.8) | 271 (41.3) | 670 (13.5) |
| **Mean total memory score (SD)** | 11.0 (3.5) | 10.1 (3.8) | 11.1 (3.4) |
| **Waist circumference** |  |  |  |
| Low | 1,315 (23.5) | 137 (20.9) | 1,178 (23.8) |
| Medium | 1,429 (25.5) | 139 (21.2) | 1,290 (26.1) |
| High | 2,863 (51.1) | 381 (58.0) | 2,482 (50.1) |

^* ‘^Depression’ = CES-D score of 4 or more, and ‘No depression’ = CES-D score of <4; ^ꝉ^US Institute of Medicine.

Supplementary Table 2. Characteristics in the analytical sample of 5,607 participants aged 50 years and older by sex from the English Longitudinal Study of Ageing (2012-2013)

|  | All participants  n (%) | Males (N=2,525)  n (%) | Females (N=3,082)  n (%) |
| --- | --- | --- | --- |
| **Depression** |  |  |  |
| Yes | 657 (11.7) | 229 (9.1) | 428 (13.9) |
| No | 4,950 (88.3) | 2,296 (90.9) | 2,654 (86.1) |
| **Vitamin D (25OHD) quartile** |  |  |  |
| Lowest | 1,406 (25.1) | 579 (22.9) | 827 (26.8) |
| Second lowest | 1,400 (25.0) | 655 (25.9) | 745 (24.2) |
| Second highest | 1,436 (25.6) | 669 (26.5) | 767 (24.9) |
| Highest | 1,365 (24.3) | 622 (24.6) | 743 (24.1) |
| **IOM^ꝉ^ vitamin D (25OHD) cut points** |  |  |  |
| >50nmol/L | 2,435 (43.4) | 1,122 (44.4) | 1,313 (42.3) |
| 30-50nmol/L | 1,847 (32.9) | 861 (34.1) | 986 (32.0) |
| <30nmol/L | 1,325 (23.6) | 542 (21.5) | 783 (25.4) |
| **Truncated vitamin D (25OHD) cut points** |  |  |  |
| >50nmol/L | 2,435 (43.4) | 1,122 (44.4) | 1,313 (42.3) |
| ≤50nmol/L | 3,172 (56.6) | 1,403 (55.6) | 1,769 (57.7) |
| **Sex** |  |  |  |
| Male | 2,525 (45.0) | - | - |
| Female | 3,082 (55.0) | - | - |
| **Age group** |  |  |  |
| 50-59 years | 1,302 (23.2) | 597 (23.6) | 705 (22.9) |
| 60-69 years | 2,283 (40.7) | 1,034 (41.0) | 1,249 (40.5) |
| 70-79 years | 1,491 (26.6) | 659 (26.1) | 832 (27.0) |
| 80+ years | 531 (9.5) | 235 (9.3) | 296 (9.6) |
| **Season of blood collection** |  |  |  |
| Winter | 1,482 (26.4) | 674 (26.7) | 808 (26.2) |
| Spring | 416 (7.4) | 183 (7.3) | 233 (7.6) |
| Summer | 1,324 (23.6) | 582 (23.1) | 742 (24.1) |
| Autumn | 2,385 (42.5) | 1,086 (43.0) | 1,299 (42.2) |
| **Wealth quintile** |  |  |  |
| Lowest quintile | 821 (14.6) | 331 (13.1) | 490 (15.9) |
| 2^nd^ quintile | 1,041 (18.6) | 429 (17.0) | 612 (19.9) |
| 3^rd^ quintile | 1,200 (21.4) | 540 (21.4) | 660 (21.4) |
| 4^th^ quintile | 1,252 (22.3) | 598 (23.7) | 654 (21.2) |
| Highest quintile | 1,293 (23.1) | 627 (24.8) | 666 (21.6) |
| **Smoking status** |  |  |  |
| Non-smoker | 2,156 (38.5) | 820 (32.5) | 1,336 (43.4) |
| Former smoker | 2,822 (50.3) | 1,422 (56.3) | 1,400 (45.4) |
| Current smoker | 629 (11.2) | 283 (11.2) | 346 (11.2) |
| **Physical activity** |  |  |  |
| Sedentary | 226 (4.0) | 95 (3.8) | 131 (4.3) |
| Low | 812 (14.5) | 271 (10.7) | 541 (17.6) |
| Moderate | 2,729 (48.7) | 1,188 (47.1) | 1,541 (50.0) |
| High | 1,840 (32.8) | 971 (38.5) | 869 (28.2) |
| **Number of cardiovascular conditions** |  |  |  |
| None | 2,166 (38.6) | 951 (37.7) | 1,215 (39.4) |
| 1 | 1,695 (30.2) | 732 (29.0) | 963 (31.3) |
| 2+ | 1,746 (31.1) | 842 (33.4) | 904 (29.3) |
| **Number of non-cardiovascular chronic conditions** |  |  |  |
| None | 2,459 (43.9) | 1,335 (52.9) | 1,124 (36.5) |
| 1 | 2,094 (37.4) | 858 (34.0) | 1,236 (40.1) |
| 2+ | 1,054 (18.8) | 332 (13.2) | 722 (23.4) |
| **Difficulties in activities of daily living (ADL)** |  |  |  |
| None | 4,779 (85.2) | 2,177 (86.2) | 2,602 (84.4) |
| 1+ | 828 (14.8) | 348 (13.8) | 480 (15.6) |
| **Difficulties in instrumental activities of daily living (IADL)** |  |  |  |
| None | 4,666 (83.2) | 2,166 (85.8) | 2,500 (81.1) |
| 1+ | 941 (16.8) | 359 (14.2) | 582 (18.9) |
| **Mean total memory score (SD)** | 11.0 (3.5) | 10.6 (3.3) | 11.3 (3.5) |
| **Waist circumference** |  |  |  |
| Low | 1,315 (23.5) | 681 (27.0) | 634 (20.6) |
| Medium | 1,429 (25.5) | 729 (28.9) | 700 (22.7) |
| High | 2,863 (51.1) | 1,115 (44.2) | 1,748 (56.7) |

^ꝉ^US Institute of Medicine
